# HPV type infection in different anogenital sites among HIV-positive Brazilian women

**DOI:** 10.1186/1750-9378-3-5

**Published:** 2008-03-14

**Authors:** Maria Alice G Gonçalves, Giorgia Randi, Annie Arslan, Luisa L Villa, Marcelo N Burattini, Silvia Franceschi, Eduardo Antonio Donadi, Eduardo Massad

**Affiliations:** 1 Division of Clinical Immunology, University of São Paulo, São Paulo, Brazil; 2 Istituto di Ricerche Farmacologiche "Mario Negri", Milan, Italy; 3 International Agency for Research on Cancer, Lyon, France; 4 Department of Virology, Ludwig Institute for Cancer Research, São Paulo, Brazil; 5 School of Medicine, Department of Pathology, University of São Paulo, São Paulo, Brazil

## Abstract

**Objectives:**

To evaluate the prevalence of human papillomavirus (HPV) types, and risk factors for HPV positivity across cervix, vagina and anus, we conducted a study among 138 women with human immunodeficiency virus (HIV).

**Goal:**

Compare the prevalence of different HPV types and the risk factors for HPV positivity in three sites.

**Results:**

The most frequently detected HPV types in all sites were, in decreasing order, HPV16, 53, 18, 61 and 81. Agreement between the cervix and vagina was good (kappa 0.60 – 0.80) for HPV16 and 53 and excellent (Kappa > 0.80) for HPV18 and 61. HPV positivity was inversely associated with age for all combinations including the anal site.

**Conclusion:**

In HIV positive women, HPV18 is the most spread HPV type found in combinations of anal and genital sites. The relationship of anal to genital infection has implications for the development of anal malignancies. Thus, the efficacy of the current HPV vaccine may be considered not only for the cervix, but also for prevention of HPV18 anal infection among immunossuppressed individuals.

## Background

More than 1.7 million people were living with human immunodeficiency virus (HIV) infection in Latin America at the end of 2004, and Brazilian women made up more than one-third (610,000) of this population [1]. Among women from 15–24 years of age, the prevalence of HIV infection in Brazil is estimated to be 0.5%. With the advent of antiretroviral drugs in Brazil, median survival time for people diagnosed with AIDS has increased from 18 to 58 months [2].

Epidemiological and laboratory data have demonstrated that persistent infection with high-risk human papillomavirus (HPV) types (like 16, 18, 31, 33, 39, 45, 52, 53, 56, 58, 59, 66, 68, 73, 82) is an essential step in the development of invasive cervical cancer and the majority of anal cancer [3]. Compared to HIV-negative women, HIV-positive women have an increased incidence and persistence of HPV infection and a concomitant increased risk of developing squamous intraepithelial lesions and cervical cancer [4-7].

In addition, compared to HIV-negative women, HIV-positive women show a higher occurrence of multiple-type HPV infection [8-10] and a relatively lower proportion of HPV16 [11]. Little information is available on the concomitant prevalence of HPV infection in different anogenital sites [12], notably in the anal area [13,14].

In this study we have compared the prevalence of different HPV types and the risk factors for HPV positivity in three sites (i.e., cervical, vaginal and anal area).

## Methods

### Patients

Study methods have been reported elsewhere [8]. Briefly, a cross-sectional study was conducted among 138 HIV-positive women, aged 19–57 (mean age = 31 years), who attended the Center of Reference in AIDS of Santos, São Paulo, Brazil between June 1996 and April 1997. Patients who reported previous hysterectomy, virgins or those who were not able to provide an informed consent form were excluded from the study. All women underwent HIV serological testing with Western blot confirmation (Cambridge Biotech Corporation, Worcester, Massachusetts, USA). CD4 cell count was performed on the date of the visit or within three months thereafter. CD4 counts were > 500 cells/mm^3 ^in 24.6% women, between 200–499 cells/mm^3 ^in 37.0%, and < 200 cells/mm^3 ^in 38.4%.

The same gynecologist applied a standard questionnaire for all patients to elicit information on sociodemographic characteristics, smoking habits, history of drug addiction, sexual behavior and use of condom. The Ethics Committee of the Medical School of the University of São Paulo, Brazil, approved the study protocol, and all participants signed informed consent forms.

### Sample collection

The same gynecologist collected exfoliated cells from 1) ectocervix, 2) vagina and 3) anal area, in that order, with separate sterile brushes, and stored in separate flasks in phosphate-buffered saline solution (PBS). To obtain samples for anal collection, individual disposable brushes moistened in sterile PBS were inserted into the anal canal and were rotated 360° as they were withdrawn.

### HPV detection and typing

After collection, cells remaining in the collection devices were washed out into a sterile buffered solution and kept at -20°C for HPV analysis. Beta-globin amplification was used to assess specimen adequacy for HPV DNA detection. Polymerase chain reaction (PCR)-based assays were carried out in the Laboratorio de Patologia e Hemoterapia do Hospital do Cancer, Fundação Antonio Prudente, São Paulo, Brazil [15].

PCR-positive samples were further typed by restriction fragment length polymorphism according to Bernard *et al*. [16], allowing the recognition of more than 42 HPV types (HPV6,11, 13, 16, 18, 26, 31, 32, 33, 34, 35, 39, 40, 42, 44, 45, 51, 52, 53, 54, 55, 56, 57, 58, 59, 61, 62, 64, 66, 67, 68, 69, 70, 71, 72, 73, 81, 82, 83, 84, LVX100 and CP6108). The following restriction enzymes were used: Bam HI, Dde I, Hae III, Hinf I, Pst I, Rsa I and Sau 3a I (Gibco BRL-Life Technologies, Los Angeles, California, USA). The identification of HPV types was performed by comparing the pattern of amplified bands with those attributed to each virus type. HPV types were classified as high-risk (or probably high-risk) and low-risk types as in Muñoz *et al*. [17]. Other HPV types that could not be identified in this study were considered unidentified (*HPVX).*

### Statistical analysis

The Kappa statistic was used to calculate the agreement between HPV positivity at the cervical *vs *vaginal or anal sites. The concordance rate was considered fair to good if Kappa values were between 0.60–0.80, and excellent if greater than 0.80. Agreement for any HPV type is defined as presence of at least one common HPV type in the 2 or 3 combined sites. ORs for detection of HPV infection and corresponding 95% CIs were calculated separately for each anogenital site and for each combination of 2 or 3 sites by means of unconditional regression equations adjusted for age (< 25, 25–34, ≥ 35). The analyses were repeated with adjustment for age and CD4 count (< 200, 200–499, ≥ 500).

## Results

### HPV prevalence by anogenital site

The prevalence of HPV infection among HIV-positive women did not differ significantly between the three anogenital sites under study. The prevalence was 61.6% in the cervix, 65.9% in the vagina, and 63.7% in the anal area. The same similarity is seen when high-risk HPV types are considered, in the cervix (36.2%), vagina (42.8%) and the anal area (43.1%). In respect to low-risk HPV types, the prevalence was 34.1% in the cervix, 35.5% in the vagina and 33.3% in the anal area. Multiple HPV infections occurred in 41.2% of HPV-positive cervical samples, 48.4% of HPV-positive vaginal samples and 44.6% of HPV-positive anal samples. The prevalence of HPV types according to the risk category in the studied female population was described elsewhere [18].

In the cervix, the most frequent high-risk HPV types were HPV16 and 53 (n = 13 each type) and HPV18 (n = 12) (Table 1). HPV61 and 81 were the most frequent low-risk HPV types (n = 11 and 9 respectively). For 9.4% of the samples the HPV type could not be characterized.

**Table 1:** Prevalence of 23 HPV types by collected site among 138 HIV-positive women. Santos, São Paulo, Brazil, 1996–1997

	**Site**								
	
	**Cervix**			**Vagina**			**Anal^a^**		
	
	**Single**	**Multiple**	**Total (%)**	**Single**	**Multiple**	**Total (%)**	**Single**	**Multiple**	**Total (%)**
**HPV -**			53 (38.4)			47 (34.1)			37 (36.3)
**HPV +**	50	35	85 (61.6)	47	44	91 (65.9)	36	29	65 (63.7)
**HR HPV+**	23	28	51 (37.0)	25	34	59 (42.8)	16	28	44 (43.1)
**LR HPV+**	24	23	47 (34.1)	19	30	49 (35.5)	15	20	35 (34.3)

**High-risk**									
16	7	6	13 (9.4)	6	11	17 (12.3)	4	5	9 (8.8)
18	4	8	12 (8.7)	4	8	12 (8.7)	2	10	12 (11.8)
31	0	5	5 (3.6)	1	5	6 (4.3)	2	1	3 (2.9)
33	3	4	7 (5.1)	1	7	8 (5.8)	2	4	6 (5.9)
39	0	1	1 (0.7)	1	0	1 (0.7)	0	1	1 (1.0)
45	0	2	2 (1.4)	0	2	2 (1.4)	0	1	1 (1.0)
52	0	1	1 (0.7)	0	2	2 91.4)	0	0	0 (0.0)
53	5	8	13 (9.4)	7	8	15 (10.9)	4	9	13 (12.7)
56	1	0	1 (0.7)	1	1	2 (1.4)	0	1	1 (1.0)
58	1	2	3 (2.2)	1	2	3 (2.2)	1	2	3 (2.9)
59	1	0	1 (0.7)	0	0	0 (0.0)	0	0	0 (0.00
66	0	3	3 (2.2)	0	3	3 (2.2)	0	4	4 (3.9)
68	1	1	2 (1.4)	3	1	4 (2.9)	1	0	1 (1.0)
73	0	1	1 (0.7)	0	1	1 (0.7)	0	0	0 (0.0)
82	0	2	2 (1.4)	0	2	2 (1.4)	0	0	0 (0.0)
Subtotal	23	44	67	25	53	78	16	38	54
**Low-risk**									
6	3	1	4 (2.9)	2	2	4 (2.9)	5	3	8 (7.8)
11	3	4	7 (5.1)	2	5	7 (5.1)	1	5	6 (5.5)
32	0	1	1 (0.7)	0	1	1 (0.7)	0	0	0 (0.0)
34	1	0	1 (0.7)	1	0	1 (0.7)	1	0	1 (1.0)
40	0	2	2 (1.4)	2	2	4 (2.9)	0	2	2 (1.8)
54	1	1	2 (1.4)	1	2	3 (2.2)	0	1	1 (1.0)
61	4	7	11 (8.0)	3	8	11 (8.0)	2	6	8 (7.3)
62	0	1	1 (0.7)	0	1	1 (0.7)	2	1	3 (2.7)
64	0	1	1 (0.7)	0	0	0 (0.0)	1	0	1 (1.0)
67	2	0	2 (1.4)	0	1	1 (0.7)	0	0	0 (0.0)
69	2	0	2 (1.4)	2	1	3 (2.2)	1	0	1 (1.0)
70	1	0	1 (0.7)	0	1	1 (0.7)	0	0	0 (0.0)
71	2	0	2 (1.4)	1	1	2 (1.4)	1	0	1 (1.0)
72	0	2	2 (1.4)	1	3	4 (2.9)	0	1	1 (1.0)
81	2	7	9 (6.5)	2	9	11 (8.0)	0	5	5 (4.6)
83	2	4	6 (4.3)	1	3	4 (2.9)	1	2	3 (2.7)
84	1	0	1 (0.7)	1	0	1 (0.7)	0	1	1 (1.0)
Subtotal	24	31	55	19	40	59	15	27	42
**Unknown**	3	10	13 (9.4)	3	15	18 (13.0)	5	7	12 (11.8)

^a^Presented for 102 HIV-positive women. HPV = human papillomavirus; HIV = human immunodeficiency virus; HR = High-risk; LR = low-risk

Similarly, in the vagina, the most frequent high-risk HPV types detected were HPV16, 53 and 18 (n = 17, 15 and 12, respectively) (Table 1), while the most frequent low-risk HPV types were HPV61 and 81 (n = 11 for each). Thirteen % of vaginal samples were HPV positive but the HPV type could not be characterized.

In the anal area, the most frequently detected high-risk HPV types were, in decreasing order, HPV53 (n = 13), 18 (n = 12), and 16 (n = 9). The most frequent low-risk HPV types were HPV 61 and 6 (n = 8 for each). In 11.8% of anal samples the HPV type could not be characterized. HPV6, but not HPV11, tended to be more frequent in the anal area than in the cervix or vagina (7.8% in the anal area *vs *2.9% in each other site).

In respect to cervical and vaginal sites, agreement for any HPV type positivity was 78.3% (Kappa value = 0.53 for any type) and varied from 92.8% (HPV16) to 98.6% (HPV61 and HPV6). Kappa values above 0.80 were found for HPV18, 33 and 61. When cervical and anal sites were compared, agreement for positivity for any HPV type was found in 68.6% of women (Kappa value = 0.31) (Table 2). The Kappa value was particularly low for HPV16 (0.11), but was relatively elevated for HPV18 (0.69), especially concerning the combination among the three sites (0.72).

**Table 2:** Agreement of HPV type detection by collected site among 138 HIV-positive women. Santos, São Paulo, Brazil: 1996–1997

	**Cervix: Vagina**				**Cervix: Anus^a^**				**Cervix, Vagina, Anus^a^**		
	
**HPV type**	Cervix only	Vagina only	Both	Agreement (Kappa value)	Cervix only	Anal only	Both	Agreement (Kappa value)	Any of the three	All three	Agreement (Kappa value)
**16**	3	7	10	92.8 (0.63)	9	7	2	84.3 (0.11)^b^	21	2	79.4 (0.32)
**18**	2	2	10	97.1 (0.82)	2	4	8	94.1 (0.69)	14	7	92.2 (0.72)
**53**	2	4	11	95.7 (0.76)	8	9	4	83.3 (0.23)	22	4	81.4 (0.45)
**6**	1	1	3	98.6 (0.74)	0	5	3	95.1 (0.52)	8	2	94.1 (0.55)
**11**	2	2	5	97.1 (0.70)	3	5	1	92.2 (0.16)	8	1	91.2 (0.33)
**61**	1	1	10	98.6 (0.90)	6	5	3	89.2 (0.29)	13	3	89.2 (0.54)
**Any type**	12	18	73	78.3 (0.53)	17	15	50	68.6 (0.31)	83	45	59.8 (0.40)

^a^Available for 102 women only; ^b^Non-significant Kappa value. HPV = human papillomavirus; HIV = human immunodeficiency virus.

### Risk factors for associations between sites for any HPV positivity

As shown in Table 3, HPV positivity were inversely associated with age in three combinations of anogenital sites (cervical+anal; vaginal+anal and cervical+vaginal+anal), all of them including anal site. No association emerged in any site between HPV positivity and smoking habits, history of drug addiction, commercial sex work and condom use, and number of years since HIV diagnosis.

**Table 3:** ORs ofHPV positivity and corresponding 95% CIs according to selected characteristics among 138 HIV-positive women^a^. Santos, São Paulo, Brazil, 1996–1997

	**Cervical+Vaginal**				**Cervical+Anal^b^**				**Vaginal+Anal^b^**				**Cervical+Vaginal+Anal^b^**			
	**Tot. No**	**Pos. (%)**	**OR^c^**	**95% CI**	**Tot. No**	**Pos. (%)**	**OR^c^**	**95% CI**	**Tot. No**	**Pos. (%)**	**OR^c^**	**95% CI**	**Tot. No**	**Pos. (%)**	**OR^c^**	**95% CI**

**Age (years)**																
≥ 35	39	38.5	1		30	10.0	1		30	13.3	1		30	6.7	1	
25–34	75	49.3	1.6	0.7–3.4	55	27.3	3.4	0.9–12.8	55	34.6	3.4	1.0–11.3	55	27.3	5.3	1.1–24.8
< 25	24	58.3	2.2	0.8–6.3	17	47.1	8.0	1.7–36.8	17	41.2	4.6	1.1–19.0	17	35.3	7.6	1.3–43.8
*p for trend*			*0.12*				*0.006*				*0.03*				*0.02*	
**Tobacco smoking**																
Never smoker	44	45.5	1		32	31.3	1		32	31.3	1		32	21.9	1	
Ex-smoker	33	45.5	1.1	0.4–2.8	26	30.8	1.1	0.3–3.8	26	34.6	1.2	0.4–3.7	26	30.8	1.7	0.5–6.1
Current smoker	61	50.8	1.3	0.6–3.0	44	18.2	0.5	0.2–1.7	44	25.0	0.8	0.3–2.2	44	18.2	0.9	0.3–2.9
*p for trend*			*0.46*				*0.22*				*0.52*				*0.60*	
**Drug addict**																
Never	79	45.6	1		62	25.8	1		62	25.8	1		62	21.0	1	
Ex	36	52.8	1.3	0.6–2.8	24	29.2	1.1	0.4–3.2	24	45.8	2.2	0.8–6.2	24	29.2	1.4	0.5–4.2
Current	23	47.8	1.0	0.4–2.7	16	18.8	0.7	0.2–2.9	16	18.8	0.6	0.2–2.6	16	18.8	0.8	0.2–3.5
*p for trend*			*0.80*				*0.70*				*0.99*				*0.98*	
**Years since HIV diagnosis**																
≤ 1	54	46.3	1		40	27.5	1		40	32.5	1		40	22.5	1	
2–4	43	39.5	0.8	0.4–1.9	34	60.529.4	1.3	0.4–3.8	34	32.4	1.0	0.4–2.8	34	26.5	1.3	0.4–4.2
≥ 5	39	56.4	1.8	0.7–4.2	26	19.2	0.7	0.2–2.8	26	23.1	0.5	0.2–1.9	26	19.2	0.8	0.2–3.1
*p for trend*			*0.25*				*0.73*				*0.38*				*0.81*	
**CD4 count/mm^3^**																
≥ 500	34	41.2	1		20	15.0	1		20	15.0	1		20	10.0	1	
200–499	51	49.0	1.8	0.7–4.7	39	23.1	4.2	0.8–22.2	39	30.8	4.8	1.0–22.7	39	23.1	5.7	0.9–34.1
< 200	53	59.9	1.8	0.7–4.6	43	32.6	5.9	1.2–28.6	43	34.9	5.0	1.1–22.4	43	27.9	6.2	1.1–34.9
*p for trend*			*0.24*				*0.03*				*0.06*				*0.06*	
**Commercial sex worker**																
Never	107	46.7	1		82	24.4	1		82	26.8	1		82	20.7	1	
Ever	31	51.6	1.2	0.5–2.6	20	30.0	1.2	0.4–3.8	20	40.0	1.7	0.6–4.8	20	30.0	1.5	0.5–4.6
**Lifetime number of sexual partners**																
≤ 2	34	41.2	1		26	26.9	1		26	30.8	1		26	26.9	1	
3–5	44	52.3	1.5	0.6–3.8	35	25.7	0.9	0.3–2.9	35	25.7	0.7	0.2–2.3	35	17.1	0.5	0.1–1.8
≥ 6	60	48.3	1.2	0.5–3.0	41	24.4	0.7	0.2–2.3	41	31.7	0.8	0.3–2.6	41	24.4	0.7	0.2–2.2
*p for trend*			*0.63*				*0.32*				*0.85*				*0.53*	
**Recent sexual partners^d^**																
0	48	43.8	1		42	21.4	1		42	26.2	1		42	21.4	1	
1	74	47.3	1.0	0.5–2.1	51	25.5	0.8	0.3–2.4	51	27.5	0.8	0.3–2.0	51	19.6	0.6	0.2–1.7
≥ 2	13	76.9	3.5	0.8–15.0	9	44.4	2.2	0.5–10.7	9	55.6	2.6	0.5–12.0	9	44.4	2.0	0.4–9.5
*p for trend*			*0.21*				*0.56*				*0.52*				*0.83*	
**Condom use^d^**																
Yes	69	47.8	1		47	25.5	1		47	27.7	1		47	19.2	1	
No	20	60.0	1.7	0.6–4.8	13	38.5	2.0	0.5–7.6	13	46.2	2.4	0.7–8.8	13	38.5	2.9	0.7–11.6
**Anal intercourse**																
Never	66	48.5	1		51	19.6	1		51	19.6	1		51	19.6	1	
Ever	72	47.2	0.9	0.5–1.8	51	31.4	1.7	0.6–4.3	51	39.2	2.4	0.97–6.0	51	25.5	1.2	0.5–3.2

^a^Some figures do not add up to the total due to missing values; ^b^Presented for 102 HIV-positive women; ^c^Adjusted for age. HPV = human papillomavirus; ^d ^relative to the last 6 months preceding interview; HIV = human immunodeficiency virus; OR = odds ratio; CI = confidence intervals.

Anal intercourse showed an association of borderline statistical significance in the combination vaginal + anal for HPV positivity (OR: 2.4; 95% CI: 0.97–6.0), but not elsewhere. Nevertheless, condom use did not show associations with HPV positivity at all sites.

## Discussion

Approximately two-thirds of HIV-positive women in our study from Brazil were infected by HPV and the prevalence of HPV infection was similar in the cervix, vagina and anal area. This also held true for the prevalence of the most frequently detected individual HPV types, notably HPV11, 16, 18, 53 and 61, and for the frequency of multiple-type infections. However, the same HPV type(s) were not always present in the three examined anogenital areas and the agreement in the detection of individual types was better between the cervix and the vagina than between the cervix and the anal area. The type most frequently detected at all three sites was HPV18, mainly on account of its high prevalence in the anal area. Recently, Castle *et al*. [19] reported that some HPV types (e.g., HPV61, 71 and 72), were more frequently detected in vaginal specimens from hysterectomized women compared to those who had not undergone hysterectomy. Simultaneous HPV infection in different anogenital sites is considered a risk factor for the concomitant occurrence of anal and cervical lesions, especially in HIV-positive women [20-22].

The factors most clearly associated with HPV positivity among HIV-positive women in our study agree with findings from HIV-negative women [23,24]. Although the small number of women available did not allow most presented ORs to reach statistical significance, we did show higher HPV positivity below age 25 than at age 35 or older, as well as among women who reported two recent sexual partners or more. These findings are consistent with the report that recent sexual partners are a stronger determinant of HPV positivity than the lifetime number of sexual partners [25]. No consistent relationship between HPV positivity and smoking habits or condom use have emerged among HIV-negative women [23,24].

Two characteristics stand out as being more strongly associated with HPV positivity in the anal area than in the cervix and vagina, one being history of anal intercourse. This finding suggests that anal infection is associated among women, as it is among bisexual and homosexual men, to direct HPV exposure [22,26]. Anal infection, however, can also be detected in women who do not report anal intercourse, particularly if a concomitant infection occurs at a genital site [27,28]. The other factor that appeared more strongly associated with anal than cervical or vaginal HPV infection was low CD4 counts, thus suggesting that immunosupression may particularly facilitate HPV infection and maybe persistence in the anal area.

One of the limitations of this work is its small sample size. As a consequence, many relatively strong associations did not attain statistical significance. Furthermore, the cross-sectional design does not allow for the capture of the dynamic nature of HPV infections. One of the strengths of this study is the wide range of HPV types tested for at different anogenital sites in HIV-positive women.

In conclusion, our study showed that high prevalences of HPV infection can be found in the entire lower genital tract in HIV-positive women. In particular, the anal area may represent a reservoir of HPV infection, notably HPV6 and 18. It will thus be important to evaluate the efficacy of the current vaccine against HPV [29,30] to prevent not only HPV infection in the cervix but also in the anal area, among immunosuppressed individuals.
